# Bio-Based Polyurethane–Urea with Self-Healing and Closed-Loop Recyclability Synthesized from Renewable Carbon Dioxide and Vanillin

**DOI:** 10.3390/polym16162277

**Published:** 2024-08-10

**Authors:** Tianyi Han, Tongshuai Tian, Shan Jiang, Bo Lu

**Affiliations:** 1 School of Medical Informatics, Chongqing Medical University, Chongqing 400016, China; tyhan2002@163.com; 2 School of Materials Science and Engineering, Shanghai University, Shanghai 200444, China; tongshuaitian@shu.edu.cn (T.T.); jiangshan891@shu.edu.cn (S.J.)

**Keywords:** vanillin, carbon dioxide, polyurethane–urea, self healing, closed-loop recyclability

## Abstract

Developing recyclable and self-healing non-isocyanate polyurethane (NIPU) from renewable resources to replace traditional petroleum-based polyurethane (PU) is crucial for advancing green chemistry and sustainable development. Herein, a series of innovative cross-linked Poly(hydroxyurethane-urea)s (PHUUs) were prepared using renewable carbon dioxide (CO_2_) and vanillin, which displayed excellent thermal stability properties and solvent resistance. These PHUUs were constructed through the introduction of reversible hydrogen and imine bonds into cross-linked polymer networks, resulting in the cross-linked PHUUs exhibiting thermoplastic-like reprocessability, self healing, and closed-loop recyclability. Notably, the results indicated that the VL-TTD*-50 with remarkable hot-pressed remolding efficiency (nearly 98.0%) and self-healing efficiency (exceeding 95.0%) of tensile strength at 60 °C. Furthermore, they can be degraded in the 1M HCl and THF (*v*:*v* = 2:8) solution at room temperature, followed by regeneration without altering their original chemical structure and mechanical properties. This study presents a novel strategy for preparing cross-linked PHUUs with self-healing and closed-loop recyclability from renewable resources as sustainable alternatives for traditional petroleum-based PUs.

## 1. Introduction

Due to its outstanding performance, polyurethane (PU) is widely utilized in various applications such as textiles, construction, adhesives, transportation, medicine, and electronics [1,2,3]. Traditional PUs are prepared from isocyanates, posing potential risks to health and ecological safety [4,5]. In recent years, with the development of green chemistry, researchers have committed to developing safe, environmentally friendly, and sustainable non-isocyanate PUs. One of the most promising strategies uses five-membered cyclic carbonate (5CC) with amino groups for preparing PUs through the addition polymerization reaction [4,6,7]. Simultaneously, the most widely used method for synthesizing 5CC is the catalytic carbonization of epoxides with CO_2_ [8,9], while CO_2_ is considered a principal factor in global warming and climate change [10]. Therefore, fixing CO_2_ into PHUs by chemical methods is significant for green chemistry and sustainable development.

Currently, most polymers are produced from non-renewable fossil energy, which leads to a series of environmental issues due to the rapid depletion of fossil fuels. Using renewable resources as raw materials to prepare sustainable polymers offers an effective approach to reducing the use of fossil energy [11,12,13]. In recent years, various renewable feedstocks such as vanillin [14,15], epoxidized soybean oil [16,17], furfuryl amine [18], and limonenes [19] have been utilized to prepare bio-based NIPU. Vanillin, derived from lignin, is the only bio-based aromatic monomer that has been industrialized [20]. Therefore, it seems to be the optimal choice for preparing NIPU from vanillin. Miao et al. developed a novel structural cross-linked PHU with outstanding mechanical properties from vanilla and CO_2_ [14]. Furthermore, the hydroxyl groups and carbamate groups in cross-linked PHU networks can experience a dynamic covalent bond exchange reaction under temperature and pressure stimulation, enabling bio-based PHUs to be created with recyclability and reprocessing [21,22]. However, the physical remodeling process of PHUs is always accompanied by prolonged high temperatures, leading to the deterioration of material properties [23]. Therefore, it is necessary to design a PHU’s network structure to achieve self-healing and reprocessing under milder conditions.

Some studies introduced dynamic covalent bonds into cross-linked polymer networks to reduce the activation energy (E_a_) of polymers, thereby reducing the time and temperature of the remodeling process. A variety of dynamic covalent bonds has been used to prepare self-healing and recyclable polymers, such as disulfide [24,25,26], B-O [27], imines [28,29,30], and Diels–Alder bonds [18,31]. In recent years, several self-healing and recyclable CO_2_-based PHUs have been reported. In 2021, Yang et al. successfully synthesized a new self-healing and recyclable PHU from CO_2_ and epoxy soybean oil by introducing disulfide bonds, and they exhibited characteristic properties of thermoplastic elastomers [32]. However, a CO_2_-based cross-linked PHU with closed-loop recyclability has not been reported. Schiff bases are commonly used dynamic reversible covalent bonds, with great potential in fabricating PHUs with closed-loop recoverability due to their mild preparation conditions and instability under acidic conditions [33,34]. Zhang et al. developed a closed-loop recovery thermosetting polymer based on supramolecular interaction–imine bonds that can be degraded and recovered at room temperature [35]. Therefore, it is feasible to combine the degradability of imine bonds with a simple synthesis process of PHUs.

**Figure 1:**
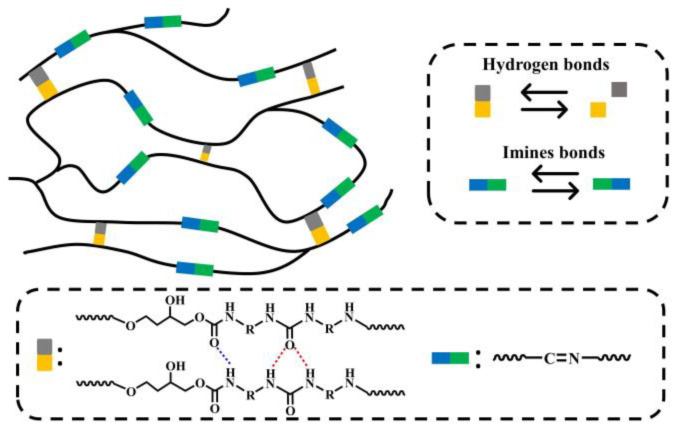
Schematic structure of VL-TTD* elastomers.

Herein, a novel cross-linked PHUU with self-healing and closed-loop recyclability was prepared by incorporating a urea-group structure and conjugated Schiff base structure into the molecular chain derived from renewable vanillin and CO_2_. This new method is more environmentally friendly than the previously reported route for preparing CO_2_-based PHUs. This study investigated the effect of the ratio of amino-terminated polyurea to a ternary amine on the thermal stability and mechanical properties of the PHUUs. Owing to the interaction between reversible imine bonds (-C=N-) and ordered/disordered hydrogen bonds in the cross-linked network (Figure 1), the PHUUs exhibited outstanding reprocessing ability and rapid self-healing. Moreover, the closed-loop recyclability of PHUUs was realized through hydrolysis and regeneration of imine bonds. This study presents an innovative and straightforward approach to developing sustainable and eco-friendly bio-based PHUUs.

## 2. Materials and Methods

### 2.1. Materials

Epichlorohydrin (ECH) and tetrabutylammonium bromide (TBAB) were provided by Aladdin Reagent Co., Ltd. Vanillin (VL), 4,7,10-Trioxa-1,13-Tridecanediamine (TTD), tris(2-aminoethyl)amine (TA), Dimethyl sulfoxide (DMSO), Tetrahydrofuran (THF), and (Methyl sulfoxide)-d6 (DMSO-d) were provided by Shanghai Titan Scientific Co., Ltd. Sodium hydroxide (NaOH), hydrochloric acid (HCl), and deuterium oxide (D_2_O) were purchased from Sinopharm Chemical Reagent Co., Ltd. (Shanghai, China). High-purity CO_2_ and nitrogen were purchased from Shanghai ChunYu Special Gas Company (Shanghai, China).

### 2.2. Synthesis of Amino-Terminal Polyurea Oligomers (TTD*)

The TTD* was synthesized from diamines (TTD) and CO_2_, and the synthesis condition and method were in accordance with our previous work [36]. In a typical experiment, 20 g TTD was placed into a 100 mL stainless-steel autoclave. The system was flushed with CO_2_ several times, and afterward, CO_2_ was introduced until pressure reached 3 MPa. Following this, the mixture was stirred for 9 h at 180 °C. A total of 23.4 g resultant amino-terminal polyurea oligomers (TTD*) was collected without additional purification. Moreover, the molecular weight of TTD* is 599 g mol^−1^.

### 2.3. Synthesis of Vanillin-Based Epoxy Monomeracetal Monomer (VL-H)

In total, 20.0 g VL, 80.0 g ECH, and 1.0 g TBAB were added to a three-necked round-bottomed flask. The solution was stirred at 80 °C for 3 h. Subsequently, the solution was cooled to 16 °C, and 13.2 g of 50 wt% aqueous sodium hydroxide was slowly added dropwise into the reaction solution over 30 min and vigorously stirred for 3 h. Then, 100 g of CH_2_Cl_2_ and 160 g of distilled water were added to the mixture and stirred for 1 h. The organic layer was poured from the mixture solution and washed five times with distilled water. After that, the organic layer was poured slowly into 300 mL of petroleum ether and vigorously stirred for 5 h. The precipitate was collected and washed three times with ethanol. The VL-H was obtained as a pale yellow powder, weighing 22.4 g, with a yield of 82.1%, after drying for 12 h in an oven at 60 °C.

### 2.4. Synthesis of Vanillin-Based 5-Membered Dicyclic Carbonates (VL-C)

The VL-C was synthesized from VL-H and CO_2_. In total, 10 g VL-H and 0.2 g TBAB were added to a 100 mL stainless-steel pressure reactor. The reactor was purged with CO_2_ five times and then heated to 150 °C for 25 min. After that, CO_2_ was introduced to the reactor with a pressure of 3.0 MPa. After stirring at 150 °C for 9 h, the reactor was cooled to room temperature to obtain the target product of VL-C (12.1 g).

### 2.5. Preparation of the VL-TTD* Networks

The cross-linked VL-TTD*s were prepared through the polyaddition reaction and Schiff base reaction of VL-C and TTD* or TA. In a typical experiment, 2.0 g VL-C, 2.1 g TTD*, 0.51 g TA, and 10.0 g DMSO were added into a three-necked round-bottom flask. The mixture was heated to 120 °C for 12 h under a nitrogen atmosphere. Subsequently, the solution was poured into a silicone rubber mold and evaporated at 60 °C for 24 h. The VL-TTD*-50 film was obtained after curing at 100 °C for 48 h in a vacuum oven. The samples were named VL-TTD*-X, where X is the molar ratio of TA, and the formulas are displayed in Table S1.

### 2.6. Characterization Methods

Nuclear magnetic resonance (^1^H NMR and ^13^C NMR) was measured on a Bruker AV-600 spectrometer (Bruker, Fällanden, Switzerland) at room temperature with deuterated chloroform (CDCl_3_) as the solvent. The ^1^H NMR curves with different temperatures were collected on a Bruker AV-400 spectrometer with DMSO-d as the solvent.

The Fourier transform infrared (FTIR) spectra were obtained using a Nicolet iS10 FTIR spectrometer (Thermo, Waltham, MA, USA) from 4000 cm^−1^ to 400 cm^−1^ equipped with attenuated total reflectance (ATR) accessories. The ATR-IR spectroscopy used a Ge prism, carrying out 16 scans, and the angle of incidence of the IR beam was 45°.

The elemental analysis (EA) was measured on a VarioEL Cubic Element Analyzer (Elementar, Langenselbold, Germany).

The solvent resistance of VL-TTD*s was determined. The rectangular samples were immersed in THF at room temperature for 48 h. Then, the swollen samples were taken out and weighed after the surface solvent was wiped. The swelling rate of VL-TTD*s was calculated as (M1 − M0)/M0, where M0 is the weight of the original sample and M1 is the weight of the swollen sample.

The gel contents of VL-TTD*s were determined using a solvent-soaking method. Around 500 mg of samples (m_0_) were soaked in THF for 48 h. Then, the insoluble residue samples were weighed (m_1_) after drying in a vacuum oven at 70 °C for 12 h. The gel content was calculated as m_1_/m_0_.

The contact angles of water were tested on an Attension Theta Flex device (Biolin, Göteborg, Sweden) and the liquid volume of water was 0.4 μL. After the droplets were deposited for 1 min, the measurement and the average value were taken three times.

Rheological measurement was performed using a Malvern Kinexus rheometer (Malvern, UK) using parallel plate geometry (20 mm diameter). The frequency sweep was performed from 0.1 to 100 Hz at 0.5% strain at room temperature.

Differential Scanning Calorimetry (DSC) was performed on a DSC Q2500 device (TA Instruments, New Castle, DE, USA) with a heating rate of 10 °C/min under a nitrogen atmosphere.

The thermal stability of VL-TTD*s was examined using a TGA Q550 device (TA Instruments, New Castle, DE, USA). All samples (5–10 mg) were heated from room temperature to 600 °C with a heating rate of 10 °C/min under a nitrogen atmosphere.

Tensile properties were determined on a universal tensile testing machine (Shenzhen Sans Technology Co., Ltd., Shenzhen, China) according to GB/T 1040-2006 [37] at room temperature with a strain rate of 20 mm min^−1^. The samples for the test were cut into dumbbell shapes (50 mm × 10 mm × 2 mm). Each sample was tested five times under the same conditions to obtain the average values.

The reprocessing of VL-TTD*s was carried out. The films of VL-TTD*s were cut into small pieces and placed on a plate vulcanizer for reprocessing. The samples were hot-pressed at 60 °C and 10 MPa for 5 min. After the samples were cooled to room temperature, the press was released and a recycled sample was obtained.

To examine the self-healing capability of VL-TTD*s, the film was divided into two halves, placed into a mold, and heated at 60 °C for 6 h in an oven. This self-healing process was recorded using an Olympus CX43 polarizing microscope (Olympus, Tokyo, Japan) optical microscope. The self-healing efficiency of VL-TTD*s was evaluated by measuring the change in tensile strength over time during the self-healing process.

For closed-loop recyclability of VL-TTD*s, small pieces of samples were individually placed in 100 mL bottles containing 40 mL of THF and 10 mL of aqueous 1M HCl at room temperature for 24 h. Then, the degraded solutions were poured into deionized water, and then the pH of the mixture was adjusted to 9 with 1M NaOH solution and stirred for 2 h. The solid powder was precipitated, washed twice with deionized water, and dried under vacuum at 60 °C for 12 h. The obtained russet solid powder was hot-pressed to obtain the regenerated VL-TTD*s film.

## 3. Results and Discussion

### 3.1. Synthesis and Characterization of VL-C and TTD*

Both VL-C and TTD* were synthesized from CO_2_. First, the VL-H was prepared through a condensation reaction from VL. Then, the VL-H was converted into the corresponding VL-C by reacting with CO_2_. The obtained VL-C was characterized using ^1^H NMR, ^13^C NMR, FTIR, and EA, which are presented in Figures S1–S3 and Table S2, respectively. In the ^1^H NMR spectra of VL, VL-H, and VL-C (Figure S1), the signals at 3.70 ppm and 9.78 ppm corresponded to the H atoms of the methyl group (-CH_3_) and aldehyde group (-CHO), respectively, while the peaks at 7.16–7.27 ppm belonged to the H atom of the benzene ring. Compared with VL, the proton signals associated with the epoxide groups of VL-H at 3.95–4.35 ppm and the peaks appeared at 4.20–5.05 ppm corresponded to the cyclic carbonates of VL-C. The ^13^C NMR spectrum (Figure S2) exhibited characteristic signals for -CH_3_ (55.92 ppm), -CH_2_- (73.87 ppm), benzene rings (110.26 ppm, 113.85 ppm, 125.96 ppm, 131.81 ppm, 150.62 ppm, and 154.41 ppm), -CHO (56.15 ppm), and five-membered cyclic carbonates (66.21 ppm, 68.43 ppm, and 152.60 ppm). The FTIR spectrum of VL-C is shown in Figure S3. Compared with the VL-H, the peak of C-O-C stretching vibration of epoxy groups at around 910 cm^−1^ disappeared, and a new peak assigned to the C=O stretching vibration of cyclic carbonate appeared at 1783 cm^−1^. As seen from Table S2, the test values of C, H, and N of VL-C were approximately equal to the theoretical values. In conclusion, VL-C was synthesized successfully.

### 3.2. Synthesis and Characterization of VL-TTD*s

**Scheme 1:**
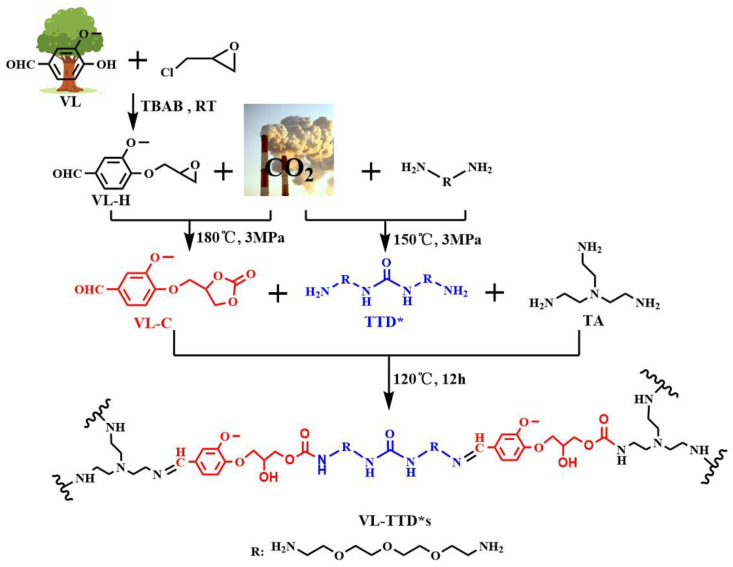
Synthetic routes of the VL-TTD*s.

**Figure 2:**
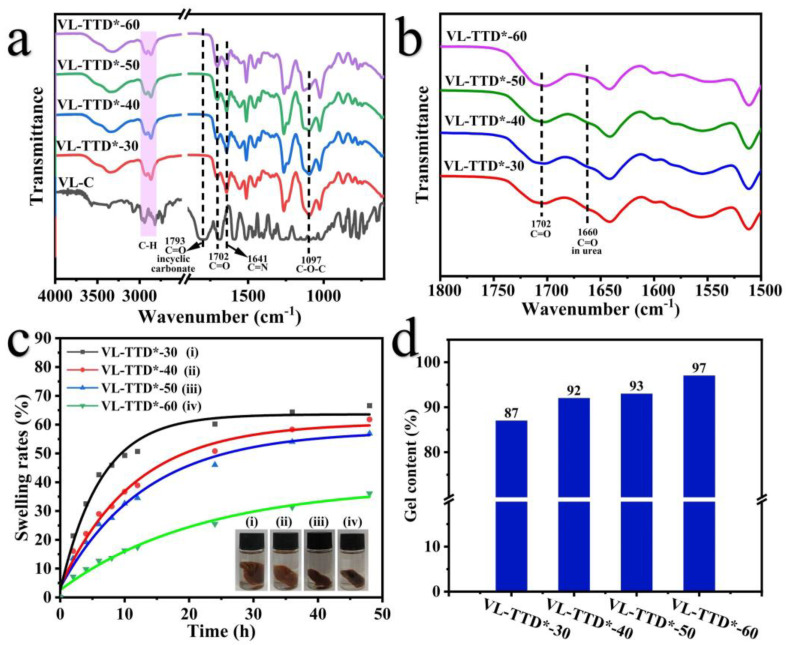
(**a**) FTIR spectra of VL−C and VL−TTD*s; (**b**) FTIR spectra of VL−TTD*s; (**c**) swelling rates of the VL−TTD*s; (**d**) gel contents of the VL−TTD*s.

To prepare a series of VL-TTD*s from VL-C, TTD* and TA were used at different amino TTD*/TA molar ratios, as presented in Scheme 1. The FTIR spectrum of VL-TTD*s can be seen in Figure 2a,b, where the peak at 3000–2800 cm^−1^ is attributed to N-H and C-H. In comparison to VL-C, the peak of C=O stretching vibration in cyclic carbonate at 1793 cm^−1^ completely disappeared, along with some new peaks of -OH, C=N, and C-O-C stretching vibrations which appeared at 3340, 1641, and 1097 cm^−1^, respectively. Additionally, the experimental values of C, H, and N in the VL-TTD*s align closely with the theoretical values (Table S2).

The solvent resistance of VL-TTD*s was assessed through a swelling experiment. The swelling rate of VL-TTD*s within 48 h and the digital photograph after being immersed in THF for 48 h at room temperature are depicted in Figure 2c. All samples exhibited remarkable solvent resistance with slight swelling after being immersed in THF for 48 h, and the swelling rates of VL-TTD*-30, VL-TTD*-40, VL-TTD*-50, and VL-TTD*-60 were 66.6%, 61.8%, 56.9%, and 36.1%, respectively. The swelling rates of VL-TTD*s decreased as the cross-linking density increased because a higher cross-linking density restricted the movement of the molecular chain. Furthermore, the gel fraction of all samples was higher than 85.0%, as shown in Figure 2d. These all indicated that the cross-linked networks of VL-TTD*s with imine bonds were successfully prepared.

**Figure 3:**
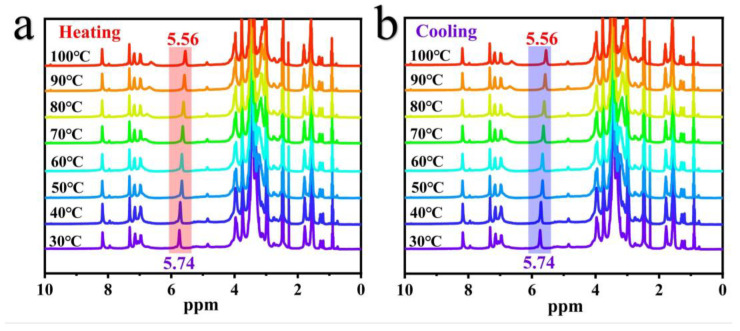
Temperature-dependent ^1^H NMR spectra of VL-TTD*-50 upon heating from 30 to 100 °C (**a**) and upon cooling from 100 to 30 °C (**b**).

The hydrogen-bonding interactions in VL-TTD*s were further demonstrated using temperature-dependent ^1^H NMR. As shown in Figure 3, the signals at 5.74 ppm corresponded to the hydrogen protons of the -NH- groups in the urea moieties [38] at 30 °C. As the temperature increased from 30 to 100 °C, the peaks of those hydrogen protons shifted to 5.56 ppm, indicating that the intermolecular hydrogen bonding was significantly disassociated. When the temperature was cooled from 100 to 30 °C, the peaks of the -NH- groups shifted from 5.56 ppm to 5.74 ppm, indicating the regeneration of intermolecular hydrogen bonds.

### 3.3. Surface Properties and Rheological Properties of VL-TTD*s

The surface hydrophobicity of the VL-TTD*s was determined by water contact angle measurement. As shown in Figure S4, the water contact angles of VL-TTD*-30, VL-TTD*-40, VL-TTD*-50, and VL-TTD*-60 are 85°, 88°, 94°, and 99°, respectively, showing a decreasing tendency with the increase in cross-linking density. This results from the molecular chains being more tightly connected as the cross-linking density increases, forming a stable three-dimensional network. Additionally, the Schiff base structure gives the PHUUs low water absorption properties.

**Figure 4:**
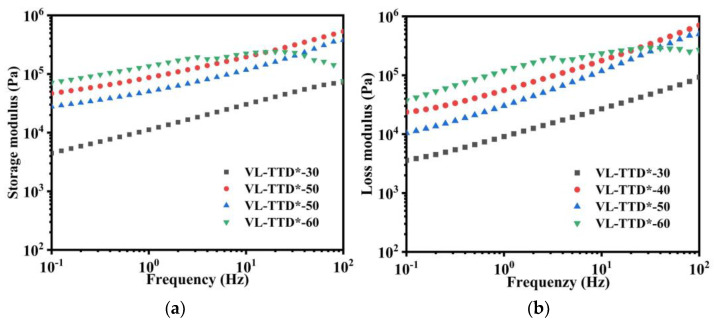
The rheology of VL-TTD*s: (**a**) Variation in the storage modulus as a function of the frequency and (**b**) variation in the loss modulus as a function of the frequency.

The rheology of the VL-TTD*s was investigated at room temperature and the results are shown in Figure 4. With an increase in cross-linking density, the storage modulus and loss modulus of VL-TTD*s showed an increasing trend. This can be attributed to the great interaction force between molecular chains resulting from higher cross-linking density. As a result, the material became more resistant to deformation under stress, leading to increased friction and energy dissipation between molecular chains. Additionally, the storage modulus and loss modulus of VL-TTD*s increased with the increase in frequency. For VL-TTD*-60, an initial increase in the loss modulus and energy storage modulus with an increase in rheological frequency was observed, followed by a decline. This is due to the molecular chain having sufficient time to respond to stress and move adequately at low frequencies. However, at high frequencies, the movement of the molecular chain became restricted, resulting in a decrease in both the energy storage modulus and the loss modulus.

### 3.4. Thermal Properties of VL-TTD*s

**Figure 5:**
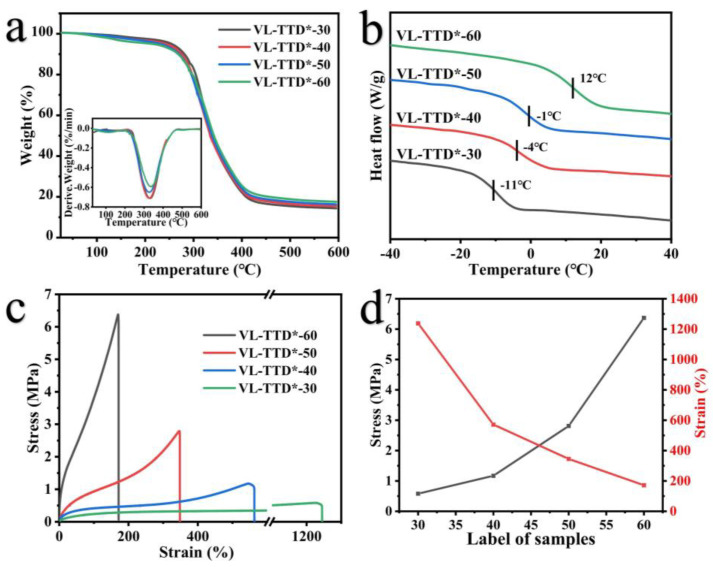
(**a**) TGA and DTG curves of VL-TTD*s in N_2_; (**b**) DSC curves of VL-TTD*s; (**c**) stress–strain curves of VL-TTD*s; (**d**) mechanical properties of VL-TTD*s.

The thermal properties of the VL-TTD*s were investigated using TGA and DSC. Figure 5a shows the TGA and DTG curves of the VL-TTD*s under an N_2_ atmosphere, and the related data are summarized in Table 1. All samples exhibited excellent thermal stability, with T_5%_ above 210 °C and T_max_ above 320 °C. Generally, as the degree of cross-linking increases, the thermal stability of a polymer is improved [39]. The strong interchain interactions were caused by the high content of urea moieties, which improved their thermal stability, but with increased cross-linking density, the urea moiety content decreased, and the T_5%_ of VL-TTD*s decreased. This proves that the influence of urea content change on the thermal stability of VL-TTD*s is greater than that of cross-linking density change. With an increase in cross-linking density, the content of the VL-TTD*s’ imide bonds increases, which is conducive to improving its high-temperature thermal stability, so T_max_ shows an increasing trend. It is worth noting that compared to VL-TTD*-30, VL-TTD*-60 showed an increased char yield from 14% to 18% at 600 °C. This can be attributed to the higher content of the -C=N- in VL-TTD*-60, which promotes charring through the cross-linking reaction among the -C=N- units at high temperatures [29,40,41]. Additionally, the second heating run of the DSC curves is depicted in Figure 5b, and the detailed data are listed in Table 1. The glass transition temperature (T_g_) of VL-TTD*-30, VL-TTD*-40, VL-TTD*-50, and VL-TTD*-60 are −11 °C, −4 °C, −1 °C, and 12 °C, respectively. The T_g_ of VL-TTD *s increased with the increase in cross-linking density.

### 3.5. Mechanical Properties of VL-TTD*s

To better understand the impact of hydrogen bond density and cross-link density on mechanical properties, the mechanical properties of VL-TTD*-30, VL-TTD*-40, VL-TTD*-50, and VL-TTD*-60 were measured by a tensile test. Generally, a PHU with higher hydrogen bonds and cross-link densities will exhibit superior thermal and mechanical properties. As shown in Figure 5c,d, as the TA content increased from 30% to 60%, the tensile strength of the VL-TTD*s rose from 0.6 MPa to 6.4 MPa, while the elongation at break decreased from 1238.0% to 171.4%. These results were attributed to the higher cross-link density restricting the movement of the molecular chains of VL-TTD*s.

### 3.6. Reprocessing Recyclability of VL-TTD*s

**Figure 6:**
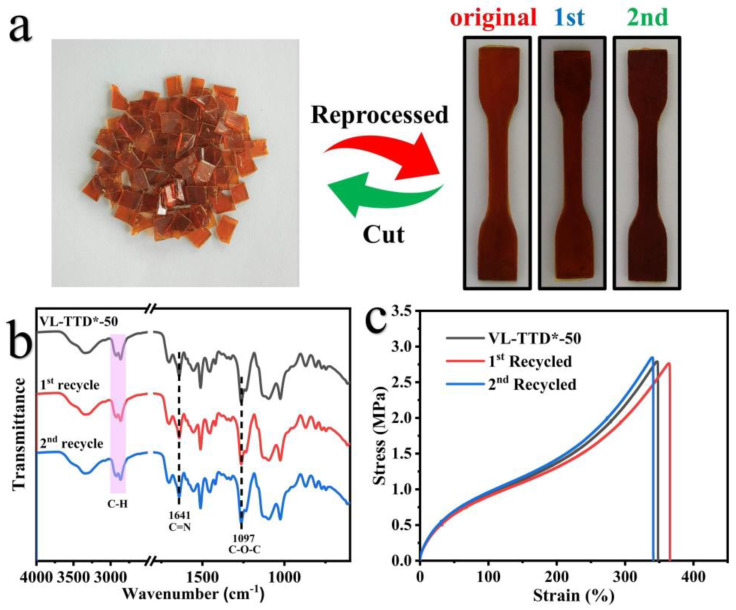
(**a**) Photographs of the hot-pressing process. (**b**) FT-IR spectra of VL-TTD*-50 after two recycling processes; (**c**) stress–strain curves of VL-TTD*-50 after two recycling processes.

There are reversible hydrogen and imine bonds in VL-TTD*s, giving them a potential physical reprocess ability. The hot-pressing process of VL-TTD*-50 is shown in Figure 6a. The film was cut into granules and hot-pressed at 60 °C and 10 MPa for 5 min using a plate vulcanizer. This recycling process was repeated twice, and the recycled samples were characterized to assess their chemical structure and mechanical properties. As shown in Figure 6b,c, the reprocessed samples displayed comparable tensile strength and elongation at break to the original ones, with the chemical structure preserved during the hot-pressing recovery process. In conclusion, the VL-TTD*s exhibited excellent stability in hot-press recycling.

### 3.7. Self-Healing of VL-TTD*s

**Figure 7:**
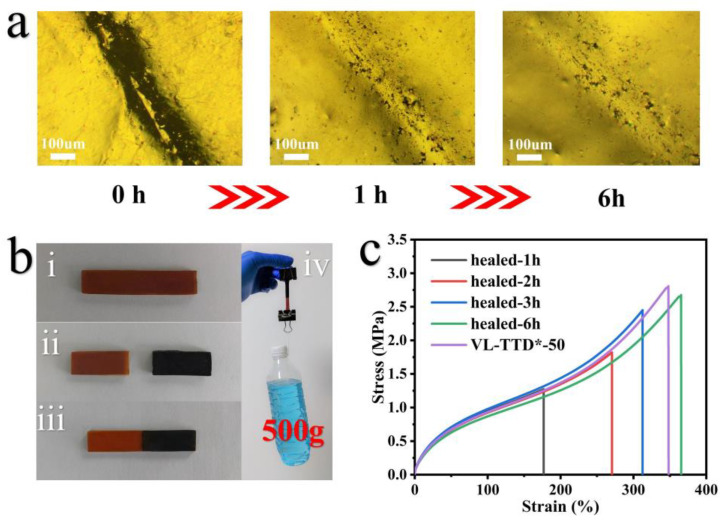
(**a**) Polarizing optical microscopy images of the self-healing process of a crack on the VL-TTD*-50; (**b**) images of the self-healing of VL-TTD*-50: (i) original sample; (ii) cut segments; (iii) healed sample; (iv) the healed sample with the hanging weight of a 500 g bottle; (**c**) stress–strain curves of the VL-TTD*-50 after healing at different times.

Due to the introduction of reversible hydrogen and imine bonds, the prepared VL-TTD*s exhibited good self-healing ability. In an experiment, the sample of VL-TTD*-50 was divided into two halves, placed into a mold, and heated at 60 °C for 6 h in an oven. This self-healing process was recorded using an optical microscope and is documented in Figure 7a. Upon heating, the cracks on the sample surface began to heal and almost disappeared after 6 h. The headed sample could easily bear a weight of 500 g without breaking, as shown in Figure 7b. The self-healing efficiency of VL-TTD*-50 was evaluated by measuring the change in tensile strength over time during the self-healing process. As presented in Figure 7c, the tensile strength of the healed sample improved with an increase in healing time, reaching 2.7 MPa and exceeding 95% healing efficiency after healing for 6 h. These results demonstrated the excellent self-healing ability of VL-TTD*s.

**Figure 8:**
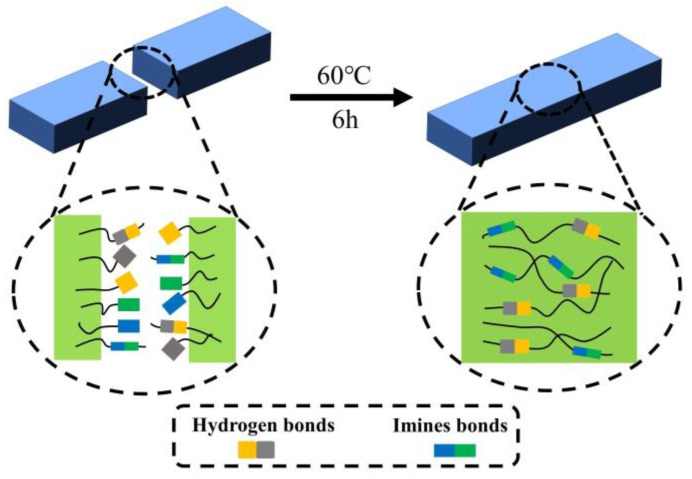
The self-healing mechanism of the VL-TTD*s.

The self-healing mechanism of VL-TTD*s is illustrated in Figure 8. The VL-TTD*s with a specific cross-linking density contain a substantial number of imine bonds and hydrogen bonds within their cross-linking networks. Imine bonds, capable of exchanging at low temperatures, facilitate rebonding at the fracture site when VL-TTD*s are damaged, thus enabling self-healing. The VL-TTD*-50 underwent heating at 60 °C, the hydrogen bond interaction in the molecular chains dissociated, and the exchange reaction of imine bonds was accelerated, leading to a more efficient self-healing process. The cracks could be filled with the chains as they had good mobility at 60 °C. Subsequently, after the cracked samples were closely connected and maintained at 60 °C for 6 h, the migrating molecular chains facilitated the reformation of the cross-linked network structure through the exchange reaction of the imine bond. Additionally, the relatively weak hydrogen bonds between molecular chains were also reformed. The widespread existence and easy formation of hydrogen bonds enable them to enhance the self-healing process alongside imine bonds. As a result, the cracked sample was healed with a healing efficiency exceeding 95.0%. In summary, during the self-healing process of cross-linked VL-TTD*s, both imine and hydrogen bonds work together through their synergistic effect, while the exchange reaction of imines provides the primary driving force for self-healing, rapid rearrangement, and rebonding facilitated by hydrogen bonding accelerates this process.

### 3.8. Closed-Loop Recyclability of VL-TTD*s

**Figure 9:**
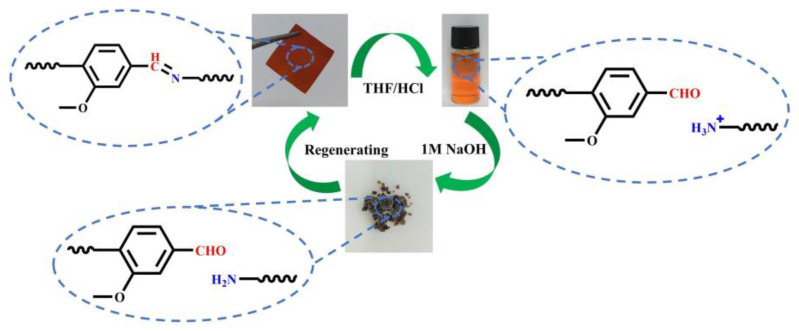
Closed-loop recycling of VL-TTD*s.

**Figure 10:**
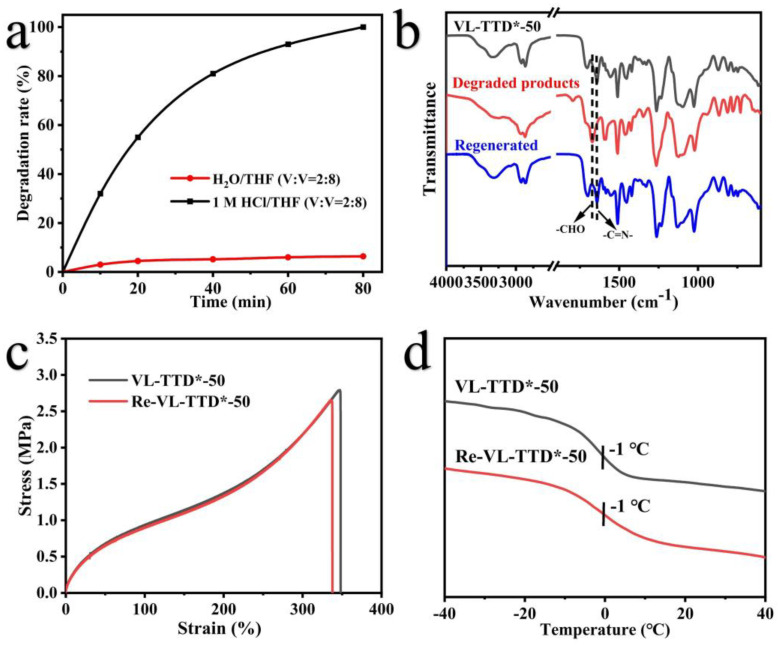
(**a**) Degradation rate curves of VL-TTD*-50 in 1M HCl and THF (*v*:*v* = 2:8) and H_2_O/THF (*v*:*v* = 2:8); (**b**) FT-IR spectra of VL-TTD*-50, degraded products, and regenerated VL-TTD*-50; (**c**) stress–strain curves of original and regenerated VL-TTD*-50; (**d**) DSC curves of original and regenerated VL-TTD*-50.

The VL-TTD*s have potential degradability attributed to the presence of imine bonds, and they can be hydrolyzed under acidic conditions [29,42]. Figure 9 shows the schematic diagram of the closed-loop recycling process and the network structure of the VL-TTD*s during the recycling process. As shown in Figure 10a, Figure S5, and Table S3, the degradation of VL-TTD*-50 was difficult in the H_2_O/THF (*v*:*v* = 2:8) solution. However, VL-TTD*-50 was completely degraded and dissolved into a mixed solution of 1M HCl and THF (*v*:*v* = 2:8) within 80 min at 25 °C, while VL-TTD*-30, VL-TTD*-40, and VL-TTD*-60 required 23 min, 61 min, and 112 min, respectively. With the increase in temperature, the degradation time of VL-TTD*-50 was shortened. When the temperature increased from 10 °C to 40 °C, the degradation time of VL-TTD*-50 was reduced from 131 min to 23 min. However, different concentrations of HCl aqueous solution did not significantly affect the degradation time when kept at the same volume ratio of 1M HCl aqueous solution with THF at 2:8. When the volume ratio of a 1M HCl aqueous solution and THF changed from 1:9 to 2:8 and 3:7, the degradation time of VL-TTD*-50 at 25 °C decreased. The structures of the degraded products were analyzed by FTIR (Figure 10b). The characteristic peaks at 1641 cm^−1^ belonging to -C=N disappeared, and a new peak assigned to -CHO groups appeared at 1673 cm^−1^, indicating the degradation of -C=N bonds.

The degraded solutions were poured into deionized water, then the pH of the mixture was adjusted to 9 with 1M NaOH solution and stirred for 2 h. The solid powder was precipitated, washed twice with deionized water, and dried under vacuum at 60 °C for 12 h. The obtained russet solid powder was hot-pressed to obtain the regenerated VL-TTD*-50 film. Additionally, the chemical structure (Figure 10b), mechanical properties (Figure 10c), and thermal properties (Figure 10d) of the regenerated sample barely changed after degradation recycling compared with the original sample.

## 4. Conclusions

In this work, we prepared a series of innovative cross-linked PHUUs with reprocessability, self-healing, and closed-loop recyclability derived from renewable CO_2_ and vanillin. The prepared VL-TTD*s exhibited excellent thermal stability properties (T_5%_ above 210 °C) and solvent resistance. Meanwhile, the mechanical properties and thermal properties of the VL-TTD*s could be tailored by adjusting the ratios of amino-terminated polyurea and ternary amine. With an increase in the cross-linking density, the tensile strength of the VL-TTD*s increased from 0.6 MPa to 6.4 MPa, accompanied by a decrease in elongation at break from 1238.0% to 171.4%. Notably, the VL-TTD*-50 exhibited remarkable hot-pressed remolding efficiency (nearly 98.0%) and self-healing efficiency (exceeding 95.0%) of tensile strength at 60 °C through the introduction of reversible hydrogen and imine bonds into the cross-linked networks. Moreover, the VL-TTD*-50 could be degraded in a mixture solution of 1M HCl/THF (*v*:*v* = 2:8) at room temperature, followed by regeneration without altering their original chemical structure and mechanical properties, achieving the closed-loop recycling of the VL-TTD*s. This work presents a novel strategy for the development of PHUUs with self-healing and closed-loop recyclability from renewable resources as substitutes for conventional PUs.

## Figures and Tables

**Table 1:** The thermal property data of VL-TTD*s.

Samples	T_5%_ (°C)	T_max_ (°C)	CR (wt%)	T_g_ (°C)
VL-TTD*-30	255	332	14	−11
VL-TTD*-40	243	326	16	−4
VL-TTD*-50	235	327	16	−1
VL-TTD*-60	216	336	18	12

T_5%_ is the temperature with 5 wt% weight loss; T_max_ is the temperature with maximum weight loss rate; CR is the char yield at 600 °C; T_g_ is the glass transition temperature.

## Data Availability

Data are contained within the article and Supplementary Materials.

## Supplementary Materials

The following supporting information can be downloaded at: https://www.mdpi.com/article/10.3390/polym16162277/s1, Figure S1. ^1^H NMR spectra of VL, VL-H, VL-C, and peak assignment; Figure S2. ^13^C NMR spectra of VL-C and peak assignment; Figure S3. FTIR spectra of VL, VC-H, and VL-C; Table S1. Formulas for the preparation of VL-TTD*s; Table S2. Elemental contents of VL-C and VL-TTD*s; Figure S4. Water contact angles of VL-TTD*s; Figure S5. Degradation process of VL-TTD*s in 1 M HCl/THF and H_2_O/THF at room temperature; Table S3. Summary of the degradation time of VL-TTD*s under different conditions.
