# Supplementary material for: Bio-Based Polyurethane–Urea with Self-Healing and Closed-Loop Recyclability Synthesized from Renewable Carbon Dioxide and Vanillin
Source: Polymers (Basel). 2024 Aug 10;16(16):2277. doi: 10.3390/polym16162277 (PMC11359345; doi:10.3390/polym16162277)
Supplement: Supplementary file 1 [file polymers-16-02277-s001.zip › polymers-3089546-supplementary.pdf]

Supporting Information

Bio-Based Polyurethane–Urea with Self-Healing and Closed-Loop Recyclability Synthesized from Renewable Carbon Dioxide and Vanillin

Tianyi Han [1,†], Tongshuai Tian [2,†], Shan Jiang [2], Bo Lu [2,]*

1 School of Medical Informatics, Chongqing Medical University, Chongqing, 400016, China; tyhan2002@163.com (T.H.).

2 School of Materials Science and Engineering, Shanghai University, Shanghai 200444, China; tongshuaitian@shu.edu.cn (T.T.); jiangshan891@shu.edu.cn(S.J.); lubo@shu.edu.cn (B.L.).

** Correspondence: lubo@shu.edu.cn (B.L.).*

† These authors contributed equally to this work.

Contents

Characterizations

Figure S1. ^{1}H NMR spectra of VL, VL-H, VL-C and peak assignment.

Figure S2. ^{13}C NMR spectra of VL-C and peak assignment.

Figure S3. FTIR spectra of VL, VC-H and VL-C.

Table S1. Formulas for the preparation of VL-TTD*s.

Table S2. Elemental contents of VL-C and VL-TTD*s

Figure S4. Water contact angles of VL-TTD*s.

Figure S5. Degradation process of VL-TTD*s in 1 M HCl/THF and H_2O/THF at room temperature.

Table S3. Summary of the Degradation Time of VL-TTD*s under Different Conditions

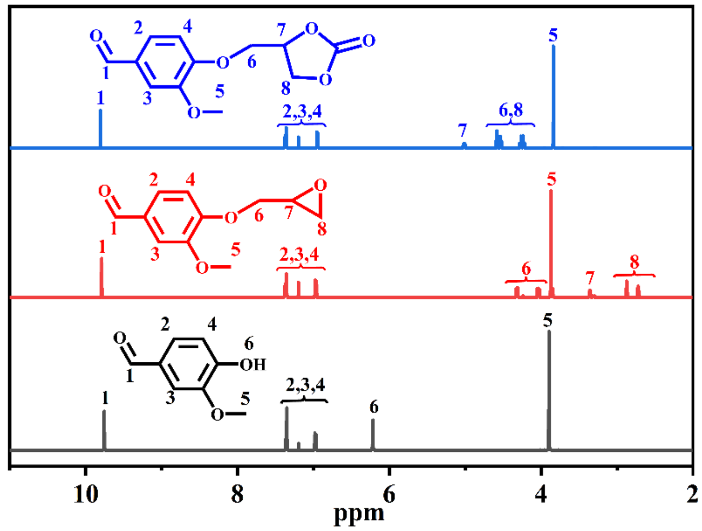

Figure S1. ^{1}H NMR spectra of VL, VL-H, VL-C and peak assignment.

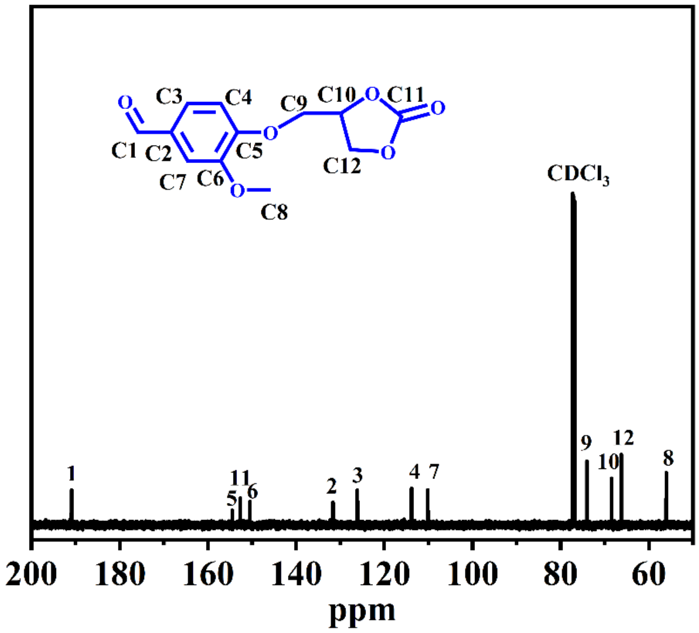

Figure S2. ^{13}C NMR spectra of VL-C and peak assignment.

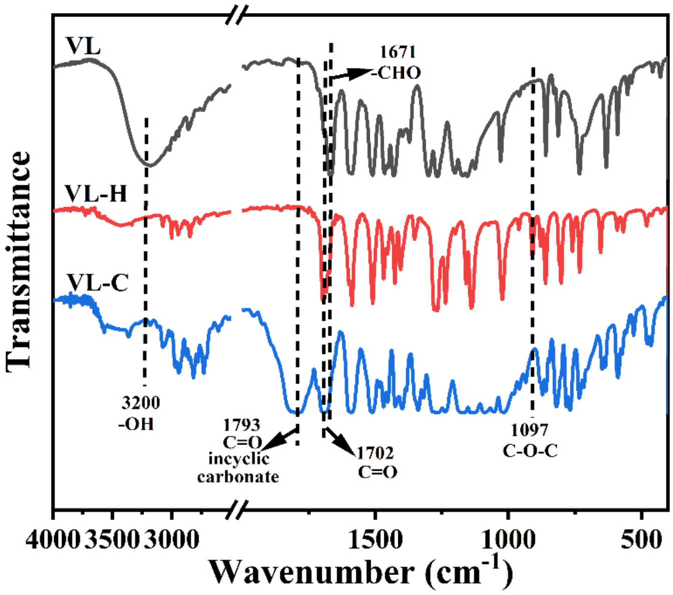

Figure S3. FTIR spectra of VL, VC-H and VL-C.

Table S1. Formulas for the preparation of VL-TTD*s.

Sample	Ratio	TA/(mol%)	TTD*/(mol%)
VL-TTD*-30	0.5	30	70
VL-TTD*-40	0.5	40	60
VL-TTD*-50	0.5	50	50
VL-TTD*-60	0.5	60	40

Ratio = the molar ratio of cyclic carbonates to amines.

Table S2. Elemental contents of VL-C and VL-TTD*s.

Samples	Theoretical value (wt%)			Test value (wt%)		
	C (%)	H (%)	N (%)	C (%)	H (%)	N (%)
VL-C	57.14	4.80	0.00	57.23	4.73	0.00
VL-TTD*-30	56.47	7.86	9.09	55.76	8.16	8.89
VL-TTD*-40	56.62	7.74	9.31	56.05	8.10	9.04
VL-TTD*-50	56.78	7.61	9.55	56.54	7.78	9.25
VL-TTD*-60	56.96	7.47	9.87	56.89	7.56	9.50

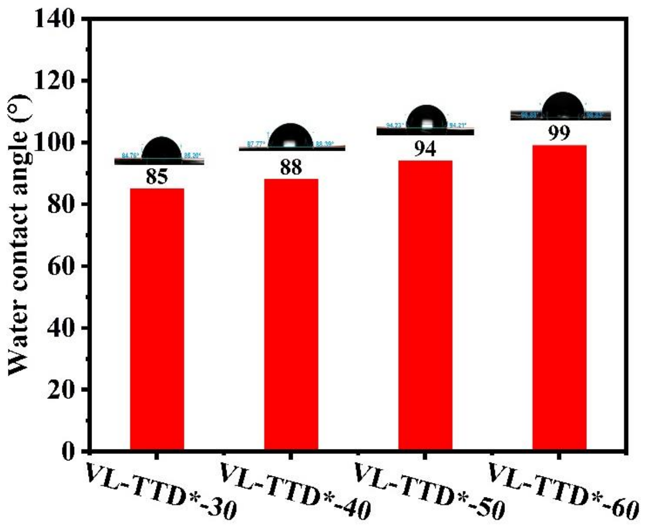

Figure S4. Water contact angles of VL-TTD*s.

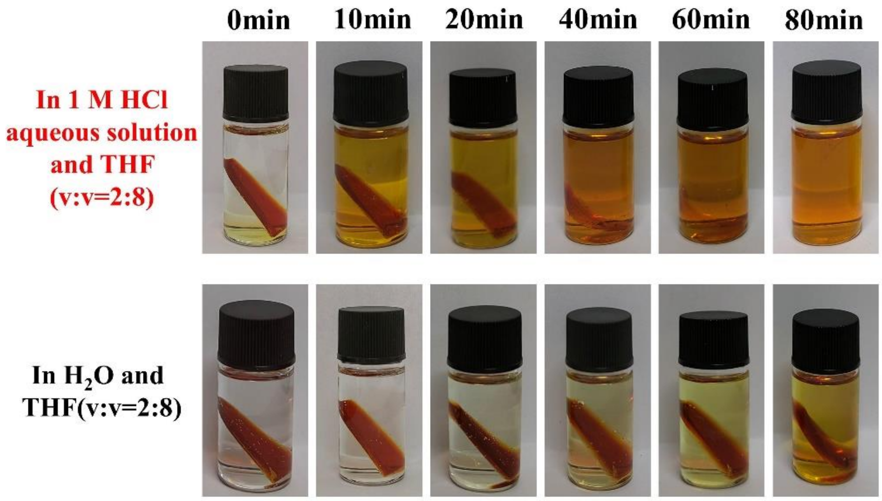

Figure S5. Degradation process of VL-TTD*s in 1 M HCl/THF and H_2O/THF at room temperature.

Table S3. Summary of the Degradation Time of VL-TTD*s under Different Conditions

Samples	Temp (℃)	THF (mL)	HCl (aq)（mL）				1.0M NaOH（aq）(mL)	Degradation time
			1.5M	1.0M	0.5M	0M		
VL-TTD*-30	25	8		2				23 min
VL-TTD*-40	25	8		2				61 min
VL-TTD*-50	25	8		2				80 min
VL-TTD*-60	25	8		2				112 min
VL-TTD*-50	25	8	2					78 min
VL-TTD*-50	25	8			2			83 min
VL-TTD*-50	25	8				0		no change after 2h
VL-TTD*-50	25	8					2	no change after 2h
VL-TTD*-50	10	8		2				131 min
VL-TTD*-50	40	8		2				28 min
VL-TTD*-50	25	9		1				87 min
VL-TTD*-50	25	7		3				75 min
